# A Brief Review of RNA–Protein Interaction Database Resources

**DOI:** 10.3390/ncrna3010006

**Published:** 2017-01-27

**Authors:** Ying Yi, Yue Zhao, Yan Huang, Dong Wang

**Affiliations:** 1 College of Bioinformatics Science and Technology, Harbin Medical University, Harbin 150081, China; lavender_yi@foxmail.com (Y.Y.); zhaoyueelf@163.com (Y.Z.); 2 Department of Child and Adolescent Health, School of Public Health, Harbin Medical University, Harbin 150081, China

**Keywords:** RNA–Protein interaction, binding site, database

## Abstract

RNA–Protein interactions play critical roles in various biological processes. By collecting and analyzing the RNA–Protein interactions and binding sites from experiments and predictions, RNA–Protein interaction databases have become an essential resource for the exploration of the transcriptional and post-transcriptional regulatory network. Here, we briefly review several widely used RNA–Protein interaction database resources developed in recent years to provide a guide of these databases. The content and major functions in databases are presented. The brief description of database helps users to quickly choose the database containing information they interested. In short, these RNA–Protein interaction database resources are continually updated, but the current state shows the efforts to identify and analyze the large amount of RNA–Protein interactions.

## 1. Introduction

RNA and protein are two important components of organisms, and the interactions between them are crucial for a large number of cellular processes, such as protein synthesis and regulation of gene expression [1,2,3,4,5,6]. In recent years, with the development of biotechnology, and especially high-throughput technology [7,8,9,10], a large number of RNA–Protein interactions have been excavated and investigated. Another rich source of high-confidence information regarding RNA–Protein interaction is the published literature. The literature in PubMed is the focus of data mining efforts to find RNA–Protein interactions. In addition, the RSCB Protein Data Bank (PDB) [11] contains direct structural verification of proteins. RNA–Protein interaction databases visualize the 3D structure of the complete interactome based on this information. Meanwhile, various computational methods are frequently used to predict both potential RNA–Protein interaction entries and the binding sites based on sequences and/or structures [12,13,14,15,16]. Some databases attempt to be comprehensive and others focus on a category of interactions. This manuscript briefly reviews some of the features of different types of interaction database resources, and it can be a guide to increase the visibility and accessibility of these resources on RNA–Protein interactions.

A list of RNA–Protein interaction database resources is given in Table 1, Table 2 and Table 3. Several widely known and used resources are listed and categorized by their contents. Table 1 describes comprehensive RNA–Protein interaction databases integrating other sources, including published literature. Table 2 lists databases containing more specialized RNA–Protein interactions that generally focus on experimental methods, or subsets of interactions, such as miRNA–Protein interactions. Table 3 shows databases consisting of the binding data of interactions with a focus on sequences and structures.

## 2. Overview of Databases

### 2.1. Comprehensive Interaction Databases

With the rapid increase of RNA–Protein interaction resources, certain interaction databases have been developed to integrate various data sources into one framework: a manual review of literature, experiments and prediction. Table 1 shows a subset of such databases.

#### 2.1.1. PRD

Protein–RNA interaction database (PRD) [17] is a database of RNA–Protein interactions database at the gene level, which integrates other data sources. The current version of PRD database contains 10,817 interaction entries, referring to 1539 unique gene pairs. It involves interactions between RNAs and proteins in 22 organisms, such as human, *Mus musculus* and *Drosophila melanogaster*, including protein-coding RNAs, tRNAs, rRNAs, miRNAs and viral RNAs. Furthermore, each interaction entry contains detailed information curated from other resources, including binding sites, Gene Ontology (GO) terms, protein/RNA motifs, detected methods and biological functions. PRD is a good reference of RNA–Protein interactions and may be helpful for the study of RNA–Protein interaction networks.

#### 2.1.2. NPInter

The noncoding RNAs and protein-related biomacromolecules interaction database (NPInter) [18] curates experimentally verified interactions between ncRNAs and other biomolecules (proteins, mRNAs, miRNAs and genomic DNAs). It is developed by Key Laboratory of RNA Biology and Beijing Key Laboratory of Noncoding RNA, Institute of Biophysics, Chinese Academy of Sciences (Beijing, China).In the newest version, NPInter documents and visualizes interactions manually collected from published literature (defined as a high-confidence set), high-throughput technologies and in silico predictions supported by high-throughput sequencing data, containing more than 491,000 interactions in 188 tissues (or cell lines), referring to 22 species (e.g., *Homo sapiens*, *Mus musculus* and *Saccharomyces cerevisiae*). Wherein, RNA–Protein interactions (more than 8000) are collected from literature mining. A local the University of California, Santa Cruz (UCSC) Genome Browser was integrated for *H. sapiens, M. musculus* and *S. cerevisiae*. Additionally, the current version provides human and mouse gene function prediction based on the interactions and gene co-expression scores between interacting molecules. The database is a helpful resource of the ncRNA interactome.

#### 2.1.3. RAID

RAID [19] is a resource of RNA-associated (RNA–Protein/RNA-RNA) interactions, developed at the Harbin Medical University (Harbin, China) and Shantou University Medical College (Shantou, China). In the current version, RAID integrates experimental and computational prediction RNA-associated interactions from 18 other resources and manually-read published literatures. It recruits 1,208,008 RNA–Protein interactions and more than 4 million RNA-RNA interactions, involves various RNAs (including circRNA, lncRNA, miRNA, mRNA, miscRNA, pseudogenes, rRNA, scRNA, sncRNA, snoRNA, snRNA, sRNA and tRNA) and contains 60 species covering seven categories (bacteria, fungi, insects, nematodes, plants, vertebrates and viruses). A confidence score was provided to evaluate the reliability of each RNA-associated interaction based on the number and type of evidence sources. RAID is a comprehensive and reliably collection of RNA-associated interactions

### 2.2. Specialized Interaction Databases

Table 2 lists several specialized interaction databases, including data derived from crosslinking immunoprecipitation (CLIP)-seq datasets (e.g., AURA [20], CLIPZ [21]) and some that have been developed as a predictive tool such as PRIdictor [22], BindN+ [23], RNABindR [24].

#### 2.2.1. CLIPdb

CLIPdb [25] is a CLIP-seq database for RNA–Protein interactions. The version has been developed to CLIPdb version 2: POSTAR [26], which is a resource of post-transcriptional regulation coordinated by RNA-binding protein (RBPs) being developed by School of Life Sciences, Tsinghua University (Beijing, China). The newest version curates a vast amount of RBP binding sites from experiments (~23 million) and predictions (~117 million) in the human and mouse transcriptomes. POSTAR provides various annotations for every transcript and its RBP binding sites, including Gene/RBP annotations, Molecular annotations, Genomic variants, Gene–Function associations and RNA secondary structures. POSTAR is the largest collection of RBP binding sites in humans and mice. It will make significant contributions to annotate post-transcriptional regulatory networks and explore the important roles of RBPs in human diseases.

#### 2.2.2. doRiNA

doRiNA [27] is a database of RNA interactions in the post-transcriptional regulation, developed at Max Planck Institute for Biology of Ageing (Cologne, Germany) and Max Delbrück Centre for Molecular Medicine (Berlin, Germany). The database contains binding site data for RNA-binding proteins and miRNAs. In the current version, RBPs target sites are identified from 136 RBP CLIP datasets for humans, mice and worms. For miRNA target sites, it presents both computational predictions (TargetScant6 and PicTar, all species) and new experimental techniques (human, mouse and *Caenorhabditis elegans*). Taking advantage of UCSC’s TrackHub feature, all target site information for RNA-binding proteins or miRNAs are integrated into the local installation of the UCSC genome browser as additional local tracks. The combinatorial action of RNA-binding proteins and miRNAs on target mRNAs form a post-transcriptional code. The database is helpful to understand the post-transcriptional regulatory network.

#### 2.2.3. starBase

starBase (sRNA target Base) [28] decodes RNA–Protein and RNA-RNA interaction network from CLIP-Seq data. It is developed by State Key Laboratory for Biocontrol, Sun Yat-sen University (Guangzhou, China). The current version contains 285,000 RNA–Protein interactions and an amount of RNA–RNA interactions identified from 108 CLIP-Seq data sets for human, mouse and *C. elegans*. Of which, the Ago protein binding sites are used to obtain CLIP-supported miRNA target sites of high confidence. In addition, miRFuction and ceRNAFunction web servers were provided to predict the function of ncRNAs and protein-coding genes based on miRNA-mediated regulatory networks. It will be a valuable resource for all RNA interactions that involve RNAs and proteins as regulators.

#### 2.2.4. RPI-Pred

RNA–Protein interaction predictor (RPI-Pred) [29] is a ncRNA–Protein interaction prediction tool based on sequence and structural information of RNA and protein. The web server can not only predict RNA–Protein interactions using the high-order structures of RNAs and proteins combined with their corresponding sequence features, but also identify the binding partners of a given RNA or protein from candidates. The performance of RPI-Pred on predicting ncRPI pairs tested across six model organisms (*C. elegans*, *D. melanogaster*, *E. coli*, *H. sapiens*, *M. musculus* and *S. cerevisiae*) shows that the tool has high prediction accuracy. RPI-Pred can be applied for developing a reliable ncRNA–Protein interaction network, which will contribute to the understanding of ncRNA’s function.

### 2.3. Binding Sites Databases

The resources in the section focus on the binding sites among RNAs and proteins based on their structures and sequences (Table 3). In databases, there are experimentally verified RNA-binding sites in proteins, and they also provide a predictive function for the potential binding sites for a given protein. Using the databases, users can not only search and browse the binding information more convenient and clearly for the known interaction pairs, but also find some potential RNA–Protein interactions.

#### 2.3.1. PRIDB

The Protein–RNA Interface Database (PRIDB) [30] is a database of RNA–Protein interfaces. PRIDB collects structural information for 926 RNA–Protein complexes in the PDB being developed at Iowa State University (Ames, USA), and includes 9689 protein chains and 2074 RNA chains. In the database, there are 1,475,774 amino acids (38% directly interact with RNA) and 851,853 ribonucleotides (28% directly interact with protein). For a RNA–Protein complex, PRIDB displays interfacial amino acids and ribonucleotides both in the protein and RNA chains and visualizes in the context of the 3D complex structure. In addition, the database can also predict the potential interface in RNA–Protein complex. PRIDB displays the information about structures of RNA–Protein complexes and their interfaces to users, and will be a reliable resource promoting the analyses of RNA–Protein interactions.

#### 2.3.2. RBPDB

The RNA-Binding Protein DataBase (RBPDB) [31] is a reservoir of experimental observation of RNA-binding sites, developed at University of Toronto (Toronto, Canada). The current version contains RNA-binding data for 1171 RBPs manually curated from published literature, referring to humans, mice, flies and worms. All data are classified by the types of RNA-binding domains. For each entry, RBPDB provides PubMed ID, the type of RNA-binding experiment, sequence and so on. Notably, users can submit a RNA/DNA sequence for potential binding sites in RNA-binding proteins stored in the database. RBPDB will be of use to diverse researchers.

#### 2.3.3. RsiteDB

The RNA binding site Data Base (RsiteDB) [32] is a database which describes, classifies and predicts the interactions between RNA nucleotide bases and protein binding pockets. The database classifies each binding site extracted from RNA–Protein complexes to the same cluster by the similarity of spatial arrangements. The clusters present physicochemical 3D consensus binding patterns. In addition, RsiteDB provides a prediction of an RNA dinucleotide binding site with a high success rate at the atomic level. It can predict its RNA binding sites and the modes of interaction when given an unbound protein structure. The classification of binding sites in RsiteDB is relevant both in the analysis of known interactions and the prediction of unknown ones.

## 3. Conclusions

Before searching for information, users want to know which database meets their needs and whether the data in the database is reliable. Such an introductory document should present the purpose of the database, the types of interactions, the data source, statistics of the data, the date of the last update and the functions provided.

The Search and Browser modules are the bridge between the data and the users. In general, the simple/advanced search and simple/advance browse panels are necessary. Users can overview the information in the database by a simple search/browse and further choose what they interested in by an advanced search/browse. The clear and well-organized presentation of search/browse results is also essential. If the number of data is large, breaking up detailed information into small pieces will be very useful. The help page is convenient for users to use the databases better. Although each database is a whole work, the correlation among databases is inevitable and important. Therefore, the extra links to other resources will help users understand data better. The process of developing and updating a database is a time-consuming and nerve-wracking task. The collection and organization of known RNA–Protein interactions is the first and most important step to establish a comprehensive interaction database. The database should provide a centralized and reliable data repository allowing users to search and browse RNA–Protein interactions systematically. Then, when data were collected from multiple resources, eliminating the heterogeneity among multiple kinds of datasets is very valuable. In the future, a comprehensive interaction databases should store more information about an interaction entry, such as a binding region/motif, structure, detection interaction method, and so on. Furthermore, the function to predict potential RNA–Protein interaction based on the sequence and structure motif in an interaction database is necessary and valuable. Given this, a user-friendly interface that provides access to databases is favorable.

In short, the complexity and diversity of cell biology can be achieved by the combinatorial possibilities offered by RNA–Protein interactome. Hence, RNA–Protein interaction databases are a necessary tool for cell biology in the future.

## Figures and Tables

**Table 1:** Comprehensive Interaction Databases.

Name	Abbr.	Version ^a^	URL	Organisms	Entries ^b^
Protein–RNA interaction database	PRD	PRD (July 2012)	http://pri.hgc.jp/	22	10,817
The noncoding RNAs and protein related biomacromolecules interaction database	NPInter	NPInter v3.0 (March 2016)	http://www.bioinfo.org/NPInter/	21	8130
RNA-associated interaction database	RAID	RAID v2.0 (October 2016)	http://www.rna-society.org/raid/	60	1,208,008

^a^ Version is the newest version of the database; ^b^ Entries represents the number of RNA–Protein interaction in database; Abbr., abbreviation.

**Table 2:** Specialized Interaction Databases.

Name	Abbr.	Version ^a^	URL	Specific	Organisms
CLIPdb	CLIPdb	CLIPdb 2: POSTAR (September 2016)	http://POSTAR.ncrnalab.org	CLIP-seq data	2 (*Homo sapiens, Mus musculus*)
doRiNA	doRiNA	DoRiNA 2.0 (October 2014)	http://dorina.mdc-berlin.de	miRNA-RBPs	4 (*H. sapiens, M. musculus, Caenorhabditis elegans* and *Drosophila melanogaster*)
sRNA target Base	starBase	starBase v2.0 (November 2013)	http://starbase.sysu.edu.cn/	CLIP-seq data	3 (Human, Mouse and *C. elegans*)
RNA–Protein interaction predictor	PRI-Pred	PRI-Pred (January 2015)	http://ctsb.is.wfubmc.edu/projects/rpi-pred	predictor	Various

^a^ Version is the newest version of the database.

**Table 3:** Binding Sites Databases.

Name	Abbr.	Version ^a^	URL	Entries ^b^
Protein–RNA Interface Database	PRIDB	PRIDB v2.0 (October 2010)	http://bindr.gdcb.iastate.edu/PRIDB	926 RNA–Protein complexes
RNA-Binding Protein Database	RBPDB	RBPDB v1.3.1 (November 2012)	http://rbpdb.ccbr.utoronto.ca/	1171 RBPs
RsiteDB	RsiteDB	RsiteDB (October 2008)	http://bioinfo3d.cs.tau.ac.il/RsiteDB	unknown

^a^ Version is the newest version of the database; ^b^ Entries represents the number of RNA-binding proteins (RBPs) or RNA–Protein complexes in database.
